# Supplementary material for: CaMK4 controls follicular helper T cell expansion and function during normal and autoimmune T-dependent B cell responses
Source: Nat Commun. 2024 Jan 29;15:840. doi: 10.1038/s41467-024-45080-x (PMC10825135; doi:10.1038/s41467-024-45080-x)
Supplement: Supplementary file 1 — Supplementary Information [file 41467_2024_45080_MOESM1_ESM.pdf]

Supplementary information

CaMK4 controls follicular helper T cell expansion and function during normal and autoimmune T-dependent B cell responses

Marc Scherlinger[1,2,3]*, Hao Li[1], Wenliang Pan[1], Wei Li[1], Kohei Karino[1], Theodoros Vichos[1], Afroditi Boulougoura[1], Nobuya Yoshida[1], Maria G. Tsokos[1] and George C. Tsokos[1]*

[1]Department of Medicine, Beth Israel Deaconess Medical Center, Boston, MA, USA.

[2] Rheumatology department, Strasbourg University Hospital of Hautepierre, Strasbourg, France.

[3] Laboratoire d'ImmunoRhumatologie Moléculaire, Institut national de la santé et de la recherche médicale (INSERM) UMR_S 1109, Strasbourg, France.

*Corresponding authors:

gtsokos@bidmc.harvard.edu and marc.scherlinger@chru-strasbourg.fr

Index:

Supplementary figures S1 to S8

Tables S1 to S3.

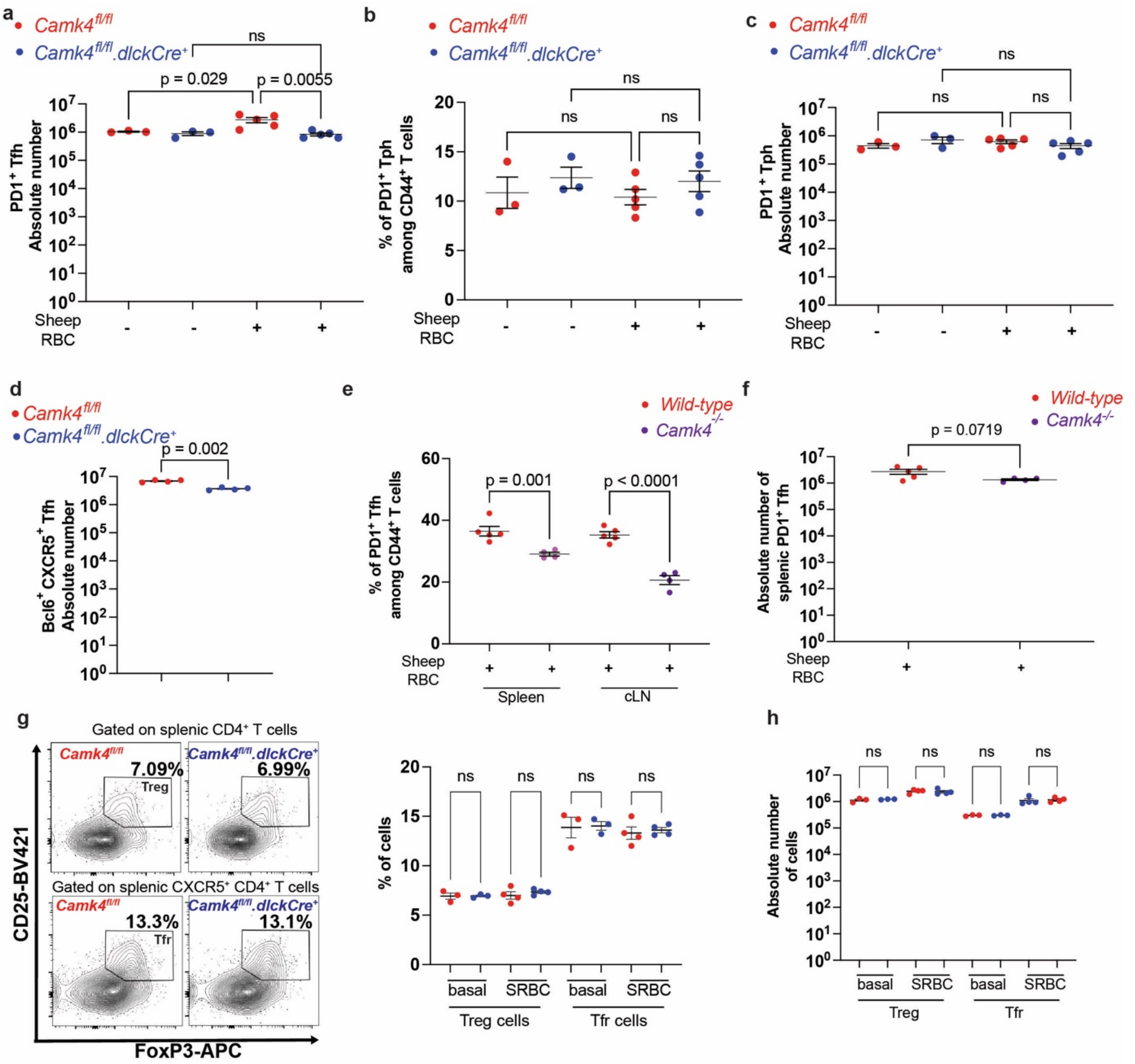

Supplementary figure 1: B6.*Camk4*$^{fl/fl}$*.dlck*^{Cre} mice immunized with sheep red blood cells have decreased Tfh expansion. (A) Absolute numbers of splenic PD1$^+$ Tfh cells. (B-C) Relative (B) and absolute (C) numbers of splenic PD1$^+$ CXCR5$^-$ T peripheral helper (Tph) cells. (D) Absolute number of splenic Bcl6$^+$ Tfh cells. (E-F) Relative (E) and absolute (F) numbers of splenic PD1$^+$ Tfh cells in wild-type (C57Bl/6) and Camk4$^{-/-}$ mice immunized with SRBC (day 7). (G) Gating strategy (left panel) and relative numbers (right panel) of T regulatory (Treg) cells at the basal state and after immunization. (H) Absolute numbers (right panel) of T regulatory (Treg) cells at the basal state and after immunization. Each point represents one mouse, bars indicate mean ± s.e.m. One-way ANOVA with Holm-Sidak's correction.

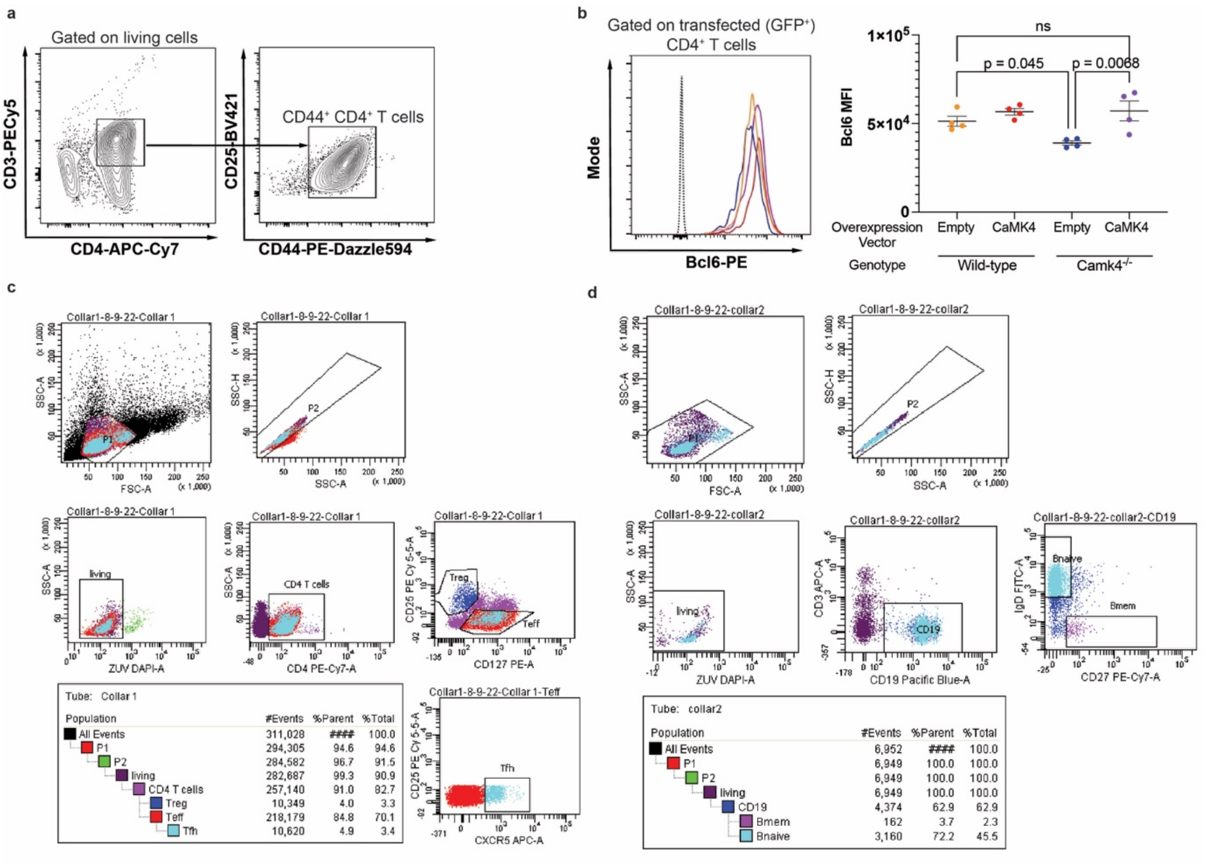

Supplementary figure 2: Gating strategy for figure 2.

(A) Gating strategy of iTfh cells. **(B)** CD62L+ CD4+ T cells from Wild-type or Camk4-/- mice (n = 4 mice per group) were differentiated to Tfh cells in vitro and transfected with an empty or CaMK4 overexpression vector. At day 3 of differentiation, Bcl6 expression was evaluated in transfected (GFP+) CD4+ T cells. One-way ANOVA with Holm-Sidak's correction. **(C)** $CD4^+$ T cells were presorted from a blood collar and Tfh were isolated using FACS as shown. **(D)** B cells were enriched from a blood collar and naïve and memory B cells were isolated as shown.

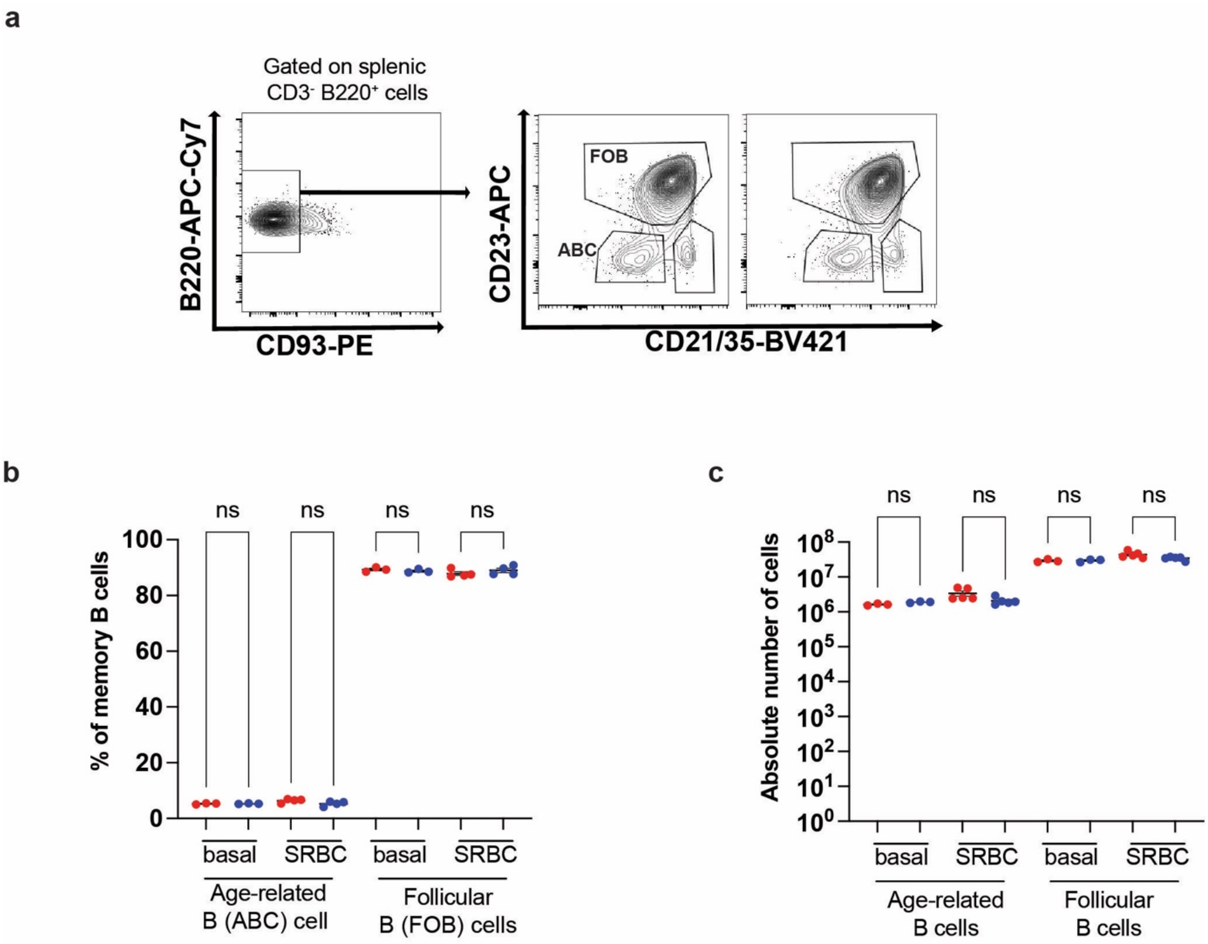

Supplementary figure 3: T-cell *Camk4* deletion does not affect other B cell subsets before and after SRBC immunization. (A) Gating strategy of B cells subtypes in the SRBC immunization model. **(B)** Relative number of B cell subtypes in the SRBC immunization model. **(C)** Absolute number of splenic B cell subtypes. Each point represents one mouse, bars indicate mean ± s.e.m. One-way ANOVA with Holm-Sidak's correction.

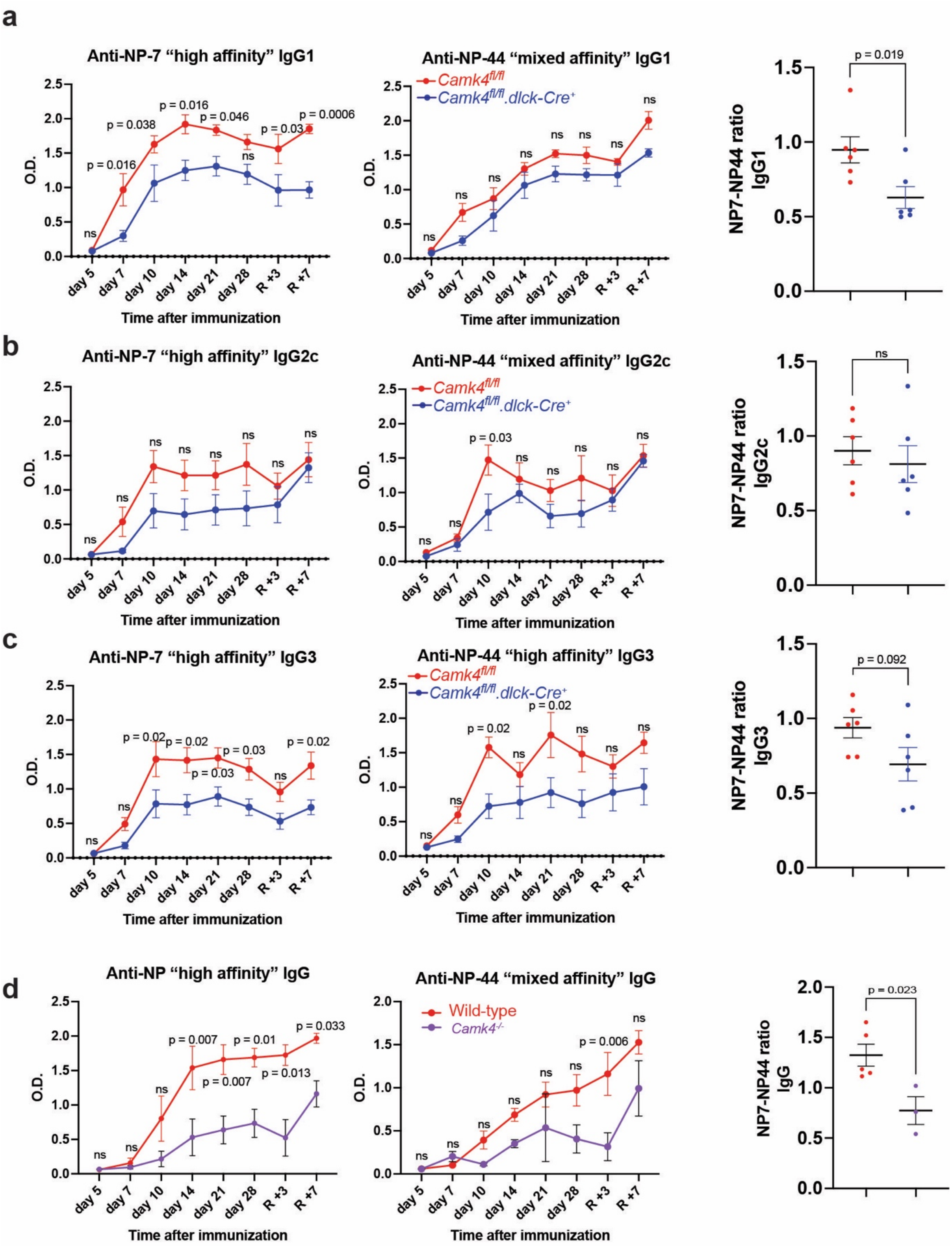

Supplementary figure 4: Impact of T-cell or global *Camk4* deletion on NP-specific IgG subsets response. *Camk4* $^{fl/fl}$ and *Camk4* $^{fl/fl}$*.dlck-Cre* mice (n = 6 per group) were immunized with NP-CGG and bled at different time point to evaluate the antibody response. ELISA results showing the optic density for the NP-7 “high affinity” IgG (left panel), NP-44 “mixed affinity” (middle panel) over time of *Camk4* $^{fl/fl}$ (red) and *Camk4* $^{fl/fl}$*.dlck-Cre* (blue) mice. The right panel indicate the NP7-NP44 ratio 7 days after recall immunization. **(A)** IgG1 results for anti-NP7 (left panel), anti-NP44 (middle panel) and the NP-7/NP44 ratio (right panel). **(B)** IgG2c results for anti-NP7 (left panel), anti-NP44 (middle panel) and the NP-7/NP44 ratio (right panel). **(C)**

IgG3 results for anti-NP7 (left panel), anti-NP44 (middle panel) and the NP-7/NP44 ratio (right panel). **(D)** Wild-type and *Camk4*$^{-/-}$ mice (n = 5 and n = 3, respectively) were immunized with NP-CGG and bled at different time point to evaluate the antibody response. ELISA results showing the optic density for the NP-7 “high affinity” IgG (left panel), NP-44 “mixed affinity” (middle panel) over time of wild-type (red) and *Camk4*$^{-/-}$ (purple) mice. Dots indicate the mean O.D. ± s.e.m. Two-way ANOVA with Holm-Sidak’s correction (longitudinal ELISA results) or Student’s t-test (NP7-NP44 ratio).

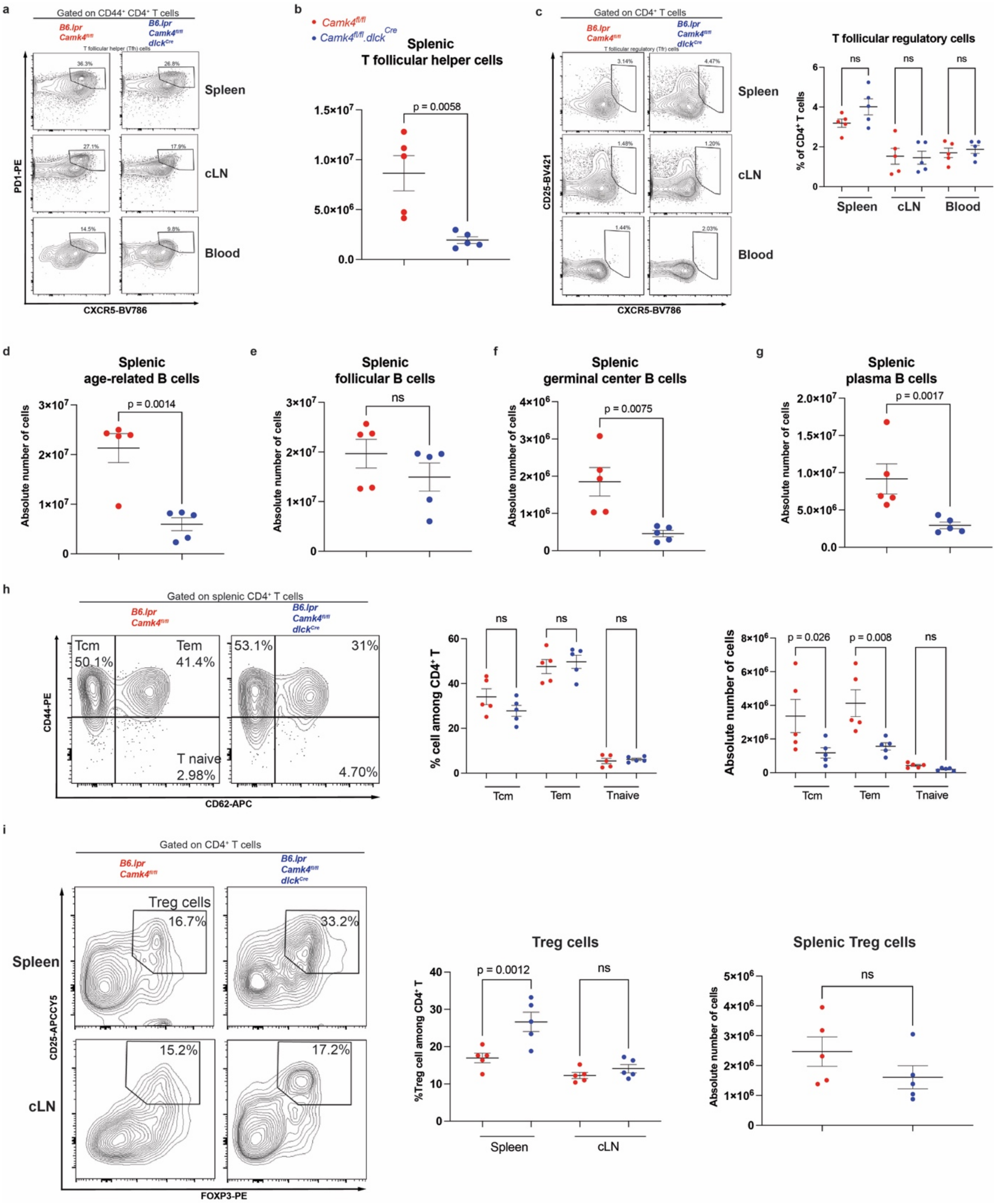

Supplementary figure 5: B6.*lpr.Camk4*$^{fl/fl}$*.dlck*^{Cre} mice have decreased Tfh cell expansion (A) Gating strategy of Tfh in the B6.lpr mice spleen, cervical lymph nodes (cLNs) and peripheral blood. (B) Absolute number of splenic Tfh cells. (C) Gating strategy (left panel) and cumulative results (right panel) of T follicular regulatory cells. (D-G) Absolute numbers of splenic age-related B cells (D), follicular B cells (E), germinal center B cells (F) and plasma cells (G). (H) Gating strategy (left panel), relative (middle panel) and absolute (right panel) of T cell subset in the spleen. (I) Gating strategy (left panel), relative (middle panel) and absolute (right panel) of T regulatory (Treg) cell in the spleen and cLNs. Each point represents one mouse, bars indicate mean ± s.e.m. One-way ANOVA with Holm-Sidak's correction (C, H, I left panel) or unpaired Student's t-test (B, D, E, F, G, I right panel).

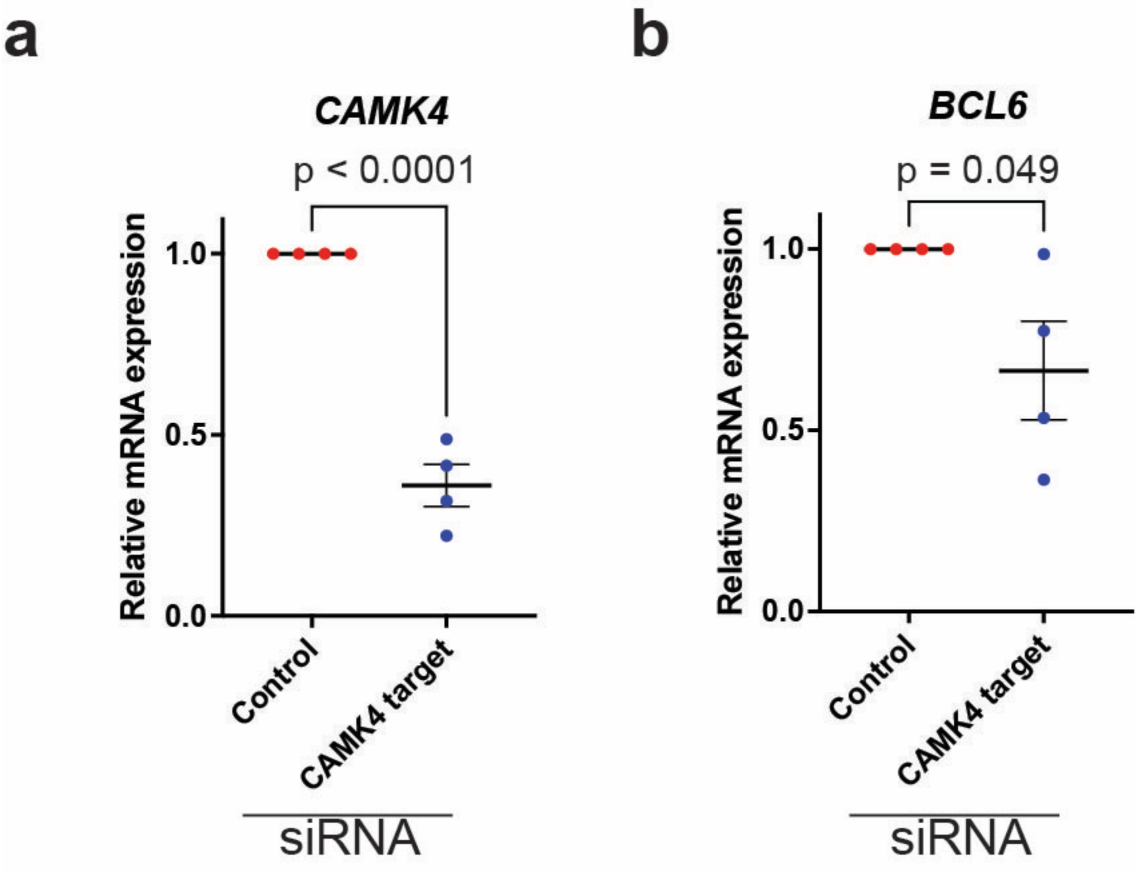

Supplementary figure 6: *CAMK4* silencing in human primary Tfh cells results in *BCL6* downregulation. Human primary $CD4^{+}CD25^{-}CD127^{+}CXCR5^{+}$ Tfh cells were sorted from healthy donors using FACS (n = 4). The cells were transfected with control or CAMK4-target siRNA. Two days after transfection, RNA was extracted,and qPCR conducted. Relative expression of CAMK4 (A) and BCL6 (B). Each dot indicates an independent experiment from a healthy donor, bars show mean ± s.e.m. Paired two-tailed Student's t-test.

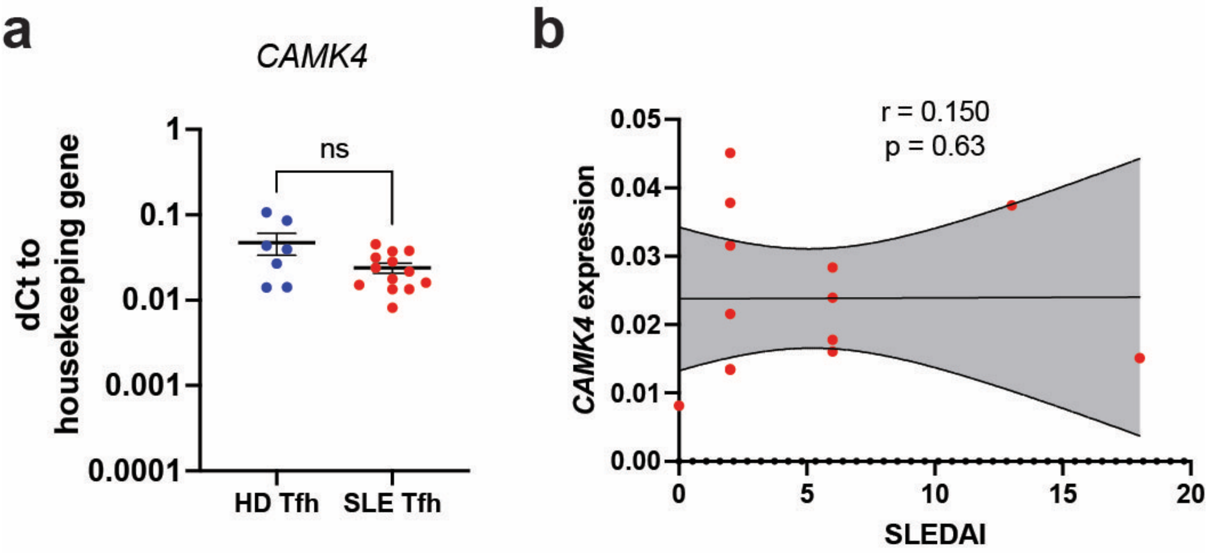

Supplementary figure 7: *CAMK4* expression in primary Tfh cells from healthy and SLE donors. (A) Comparison of CAMK4 expression using RT-qPCR in healthy donor (HD, n = 7) and SLE (n = 13) sorted primary Tfh cells. **(B)** Spearman correlation between CAMK4 expression in Tfh and the SLE disease activity index (SLEDAI; n = 13 patients). Bars show mean ± s.e.m Ns, non-significant using unpaired two-tailed Student's t-test.

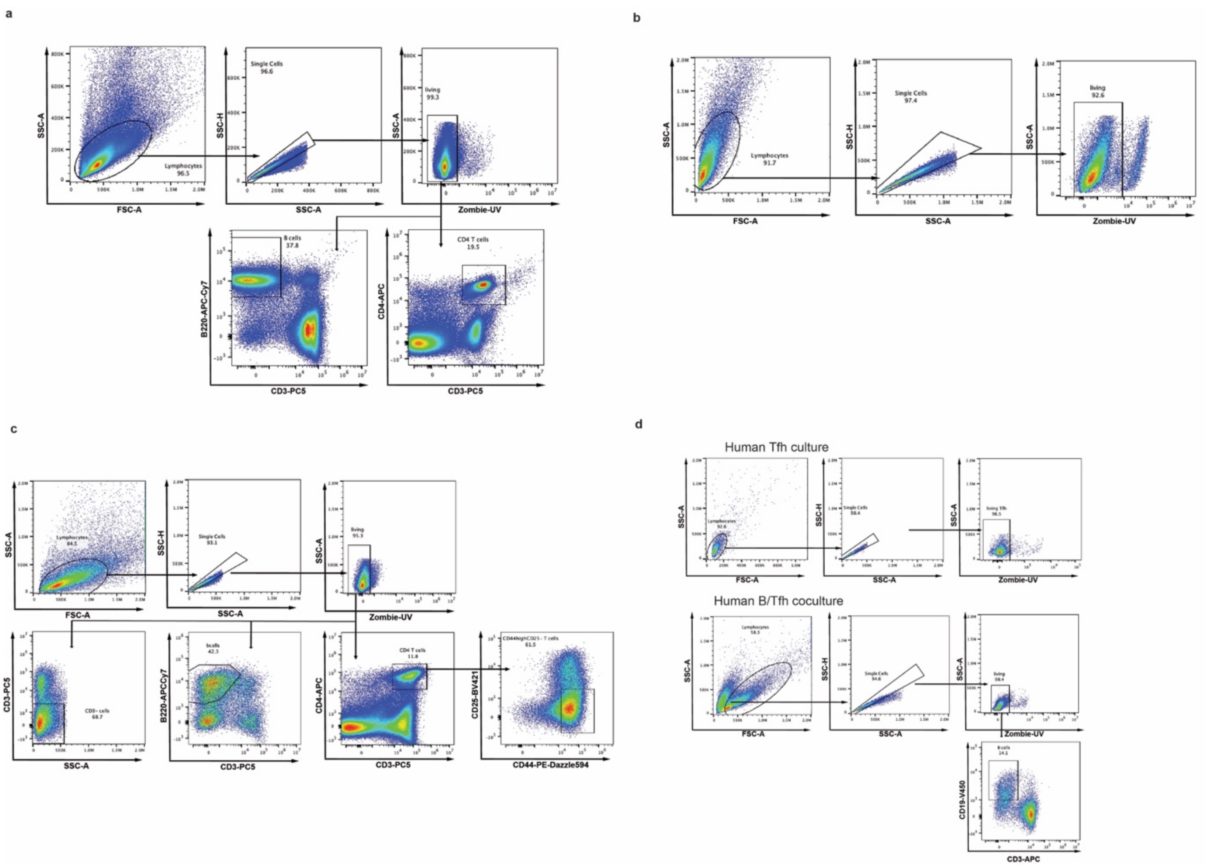

Supplementary figure 8: Gating strategies for main and supplementary figures. **(a)** Gating strategy for figures 1, 3, 4, S1 and S4. **(b)** Gating strategy for figures 2 and S2. **(c)** Gating strategy for figures 5 and S5. **(d)** Gating strategy for figure 6.

Supplementary table 1: Characteristics of systemic lupus erythematosus patients included in the study.

**Patient and disease characteristics**	**SLE patients (n = 13)**
Age, mean (S.D.), years	35.8 (11.7)
Female sex	100%
Ethnicity<br>- Caucasian<br>- Asian<br>- Hispanic<br>- Black	 23%<br>30.8%<br>15.4%<br>30.8%
Year since diagnosis, mean (S.D.), years	9.2 (6.0)
SLEDAI at inclusion, median (IQR 25-75)<br>- SLEDAI ≥ 6	2 (2-6)<br>46.1%
Autoantibodies<br>- Anti-dsDNA<br>- Anti-Sm<br>- Anti-RNP<br>- Anti-SSA<br>- Antiphospholipid (any)	 61.5%<br>30.8<br>76.9%<br>61.5%<br>38.5
Organ involvement (history of) :<br>- Skin<br>- Arthritis<br>- Nephritis<br>- Hematologic<br>- Pericarditis/pleuritis<br>- Neuropsychiatric	 76.9%<br>92.3%<br>53.8%<br>38.5%<br>23.1%<br>15.4%
Hydroxychloroquine treatment	100%
Current prednisone treatment<br>- Dose, median (IQR 25-75), mg	38.5%<br>15 (5-20)
Immunosuppressive drug<br>- Mycophenolate mofetil<br>- Azathioprine<br>- Belimumab	 69.2%<br>15.4%<br>30.8%

Supplementary table 2: Antibodies used in the study.

Antibody	Supplier	Reference	Dilution
**Murine B panel**			
B220-APCCy7	Biolegend	103224	1/200
CD21/CD35-BV421	Biolegend	123421	1/200
CD23-APC	Biolegend	101620	1/200
GL7-FITC	Biolegend	144603	1/200
CD3-PC5	Biolegend	100274	1/200
CD95-PC7	Biolegend	557653	1/200
CD93-PE	Biolegend	136503	1/200
CD138-BV605	Biolegend	142515	1/200
NP-PE	Santa Cruz	Sc-396483	1/100
CD93-BUV563	BD Bioscience	741331	1/200
**Murine Tfh panel**			
CD4-APC	Biolegend	116014	1/200
PD1-PE	Biolegend	135206	1/100
CXCR5-BV785	Biolegend	145523	1/100
CD25-BV421	Biolegend	102034	1/200
CD3-PC5	Biolegend	100274	1/200
CD44-PE-Dazzle594	Biolegend	103056	1/200
**Murine Tfh panel intracellular**			
CD4-APCCy7	Biolegend		1/200
CD3-PC5	Biolegend	100274	1/200
CD25-BV421	Biolegend	102034	1/200
CXCR5-BV785	Biolegend	145523	1/100
CD44-PE-Dazzle594	Biolegend	103056	1/200
PD1-FITC	Biolegend	135206	1/200
Bcl6-PE	Biolegend	648304	1/50
FoxP3-AF647	ThermoFisher	MA5-18160	1/50
**Sorting human Tfh**			
CD127-PE	Biolegend	351303	1/50
CD25 PE-fire700	Biolegend	356145	1/50
CD4-PC7	Biolegend	357409	1/50
CXCR5-APC	Biolegend	356906	1/50
CD19-PB	Biolegend	302224	1/50
**Sorting human B cells**			
CD3-AF647	Biolegend	300416	1/50
CD19-PB	Biolegend	302224	1/50
CD27-PC7	Biolegend	302838	1/50
IgD-FITC	Biolegend	307808	1/50
**Coculture Tfh/B cell**			
CD3-AF647	Biolegend	300416	1/100
CD19-PB	Biolegend	302224	1/100
CD27-APCCy7	Biolegend	302816	1/100
CD38-PC7	Biolegend	356505	1/100
**Human Tfh cytokine production**			
IL21-PE	Biolegend	513003	1/50
**Viability markers**			
Zombie UV	Biolegend	423108	1/500
Zombie Aqua	Biolegend	423102	1/500
**CHIP assay**			

CREMα	Santa Cruz	Sc390426	1μg per IP
Control IgG	Life Technologies	49-2024	1μg per IP

Supplementary table 3: Primers, vectors and siRNA used in the study.

Target	Reference
**Taqman murine**	
Camk4	Mm01135329_m1
Gapdh	Mm99999915_g1
Bcl6	Mm00477633_m1
**Taqman human**	
CAMK4	Hs00174318_m1
BCL6	Hs00153368_m1
PDCD1	Hs00169472_m1
IL21	Hs00222327_m1
GAPDH	Hs02758991_m1
**Luciferase assay**	
Renilla *Bcl6*	pGL3_Bcl6_vector (Genscript)
Firefly control	pGL3_firefly_vector (Genscript)
**CHIP-PCR**	
Bcl6 promoter region	Forward: 5'-CTTTGCTACAGCGAAGACGC-3'<br>Reverse: 5'-CACCGATGGAACCGCCC-3'
**Overexpression vector**	
Camk4	pcDNA3.1(+)-P2AeGFP (Genscript)
Empty vector	pcDNA3.1(+)-P2AeGFP (Genscript)
**siRNA**	
Control	ON-TARGETplus Non-targeting Control Pool (Horizon Discovery)
CAMK4 target	ON-TARGETplus Human CAMK4 siRNA SMARTPOOL (Horizon Discovery)
