# Supplementary material for: CaMK4 controls follicular helper T cell expansion and function during normal and autoimmune T-dependent B cell responses
Source: Nat Commun. 2024 Jan 29;15:840. doi: 10.1038/s41467-024-45080-x (PMC10825135; doi:10.1038/s41467-024-45080-x)
Supplement: Supplementary file 3 — Reporting Summary [file 41467_2024_45080_MOESM3_ESM.pdf]

nature portfolio

Corresponding author(s): George C. TSOKOS

Last updated by author(s): Jan 19, 2023

# Reporting Summary

Nature Portfolio wishes to improve the reproducibility of the work that we publish. This form provides structure for consistency and transparency in reporting. For further information on Nature Portfolio policies, see our Editorial Policies and the Editorial Policy Checklist.

## Statistics

For all statistical analyses, confirm that the following items are present in the figure legend, table legend, main text, or Methods section.

| n/a | Confirmed | |
|---|---|---|
| ☐ | ☒ | The exact sample size (*n*) for each experimental group/condition, given as a discrete number and unit of measurement |
| ☐ | ☒ | A statement on whether measurements were taken from distinct samples or whether the same sample was measured repeatedly |
| ☐ | ☒ | The statistical test(s) used AND whether they are one- or two-sided<br>*Only common tests should be described solely by name; describe more complex techniques in the Methods section.* |
| ☒ | ☐ | A description of all covariates tested |
| ☐ | ☒ | A description of any assumptions or corrections, such as tests of normality and adjustment for multiple comparisons |
| ☐ | ☒ | A full description of the statistical parameters including central tendency (e.g. means) or other basic estimates (e.g. regression coefficient) AND variation (e.g. standard deviation) or associated estimates of uncertainty (e.g. confidence intervals) |
| ☐ | ☒ | For null hypothesis testing, the test statistic (e.g. *F*, *t*, *r*) with confidence intervals, effect sizes, degrees of freedom and *P* value noted<br>*Give P values as exact values whenever suitable.* |
| ☒ | ☐ | For Bayesian analysis, information on the choice of priors and Markov chain Monte Carlo settings |
| ☒ | ☐ | For hierarchical and complex designs, identification of the appropriate level for tests and full reporting of outcomes |
| ☐ | ☒ | Estimates of effect sizes (e.g. Cohen's *d*, Pearson's *r*), indicating how they were calculated |

*Our web collection on statistics for biologists contains articles on many of the points above.*

## Software and code

Policy information about availability of computer code

| | |
|---|---|
| Data collection | Western blot : ImageLab 6.1.0<br>Microscropy : Fiji 2.3.0 |
| Data analysis | Prism Graphpad 10.0.2<br>Western blot : ImageLab 6.1.0 |

For manuscripts utilizing custom algorithms or software that are central to the research but not yet described in published literature, software must be made available to editors and reviewers. We strongly encourage code deposition in a community repository (e.g. GitHub). See the Nature Portfolio guidelines for submitting code & software for further information.

## Data

Policy information about availability of data

All manuscripts must include a data availability statement. This statement should provide the following information, where applicable:

- Accession codes, unique identifiers, or web links for publicly available datasets
- A description of any restrictions on data availability
- For clinical datasets or third party data, please ensure that the statement adheres to our policy

All source data are provided with the manuscript

## Human research participants

Policy information about studies involving human research participants and Sex and Gender in Research.

| | |
|---|---|
| Reporting on sex and gender | Sex is reported in our study and the patients gave consent to report this data. |
| Population characteristics | All patient data are provided in the study supplementary materials. |
| Recruitment | Patients with a diagnosis of systemic lupus fulfilling the ACR/EULAR 2019 classification criteria of SLE were recruited in the study. All the patients recruited were consulting in our lupus clinics. The vast majority of SLE patients followed in our center gave consent for research, limiting the risk for a self-selection biais. |
| Ethics oversight | The Beth Israel Deaconess Medical Center Institutional Review Board approved this study protocol.<br>All patient provided written informed consent to participate in the study. |

Note that full information on the approval of the study protocol must also be provided in the manuscript.

# Field-specific reporting

Please select the one below that is the best fit for your research. If you are not sure, read the appropriate sections before making your selection.

[x] Life sciences [ ] Behavioural & social sciences [ ] Ecological, evolutionary & environmental sciences

For a reference copy of the document with all sections, see nature.com/documents/nr-reporting-summary-flat.pdf

# Life sciences study design

All studies must disclose on these points even when the disclosure is negative.

| | |
|---|---|
| Sample size | For the lupus cohort, a number of patients of > 12 was deemed necessary to account for the disease heterogeneity. No sample size calculation was conducted but the size of the sample is similar to other research in the field.<br>All patients and cotreatment characteristics are given in Supplementary data. |
| Data exclusions | None |
| Replication | All experiments were confirmed several times on independent experiments : at least 3 independent experiments are reported for each experiment. |
| Randomization | Randomization was not applicable since the group are defined based on animal genotype. |
| Blinding | Blinding was not conducted during the study since most the experiments were conducted by a single person (MS). |

# Reporting for specific materials, systems and methods

We require information from authors about some types of materials, experimental systems and methods used in many studies. Here, indicate whether each material, system or method listed is relevant to your study. If you are not sure if a list item applies to your research, read the appropriate section before selecting a response.

### Materials & experimental systems

| n/a | Involved in the study | |
|---|---|---|
| [ ] | [x] | Antibodies |
| [x] | [ ] | Eukaryotic cell lines |
| [x] | [ ] | Palaeontology and archaeology |
| [ ] | [x] | Animals and other organisms |
| [x] | [ ] | Clinical data |
| [x] | [ ] | Dual use research of concern |

### Methods

| n/a | Involved in the study | |
|---|---|---|
| [x] | [ ] | ChIP-seq |
| [ ] | [x] | Flow cytometry |
| [x] | [ ] | MRI-based neuroimaging |

## Antibodies

| | |
|---|---|
| Antibodies used | See supplementary data<br>Antibody Brand Reference Dilution |

Murine B panel
B220-APCCy7 Biolegend 103224 1/200
CD21/CD35-BV421 Biolegend 123421 1/200
CD23-APC Biolegend 101620 1/200
GL7-FITC Biolegend 144603 1/200
CD3-PC5 Biolegend 100274 1/200
CD95-PC7 Biolegend 557653 1/200
CD93-PE Biolegend 136503 1/200
CD138-BV605 Biolegend 142515 1/200
Murine Tfh panel
CD4-APC Biolegend 116014 1/200
PD1-PE Biolegend 135206 1/100
CXCR5-BV785 Biolegend 145523 1/100
CD25-BV421 Biolegend 102034 1/200
CD3-PC5 Biolegend 100274 1/200
CD44-PE-Dazzle594 Biolegend 103056 1/200
Murine Tfh panel intracellular
CD4-APCCy7 Biolegend  1/200
CD3-PC5 Biolegend 100274 1/200
CD25-BV421 Biolegend 102034 1/200
CXCR5-BV785 Biolegend 145523 1/100
CD44-PE-Dazzle594 Biolegend 103056 1/200
PD1-FITC Biolegend 135206 1/200
Bcl6-PE Biolegend 648304 1/50
FoxP3-AF647 ThermoFisher MA5-18160 1/50
Sorting human Tfh
CD127-PE Biolegend 351303 1/50
CD25 PE-fire700 Biolegend 356145 1/50
CD4-PC7 Biolegend 357409 1/50
CXCR5-APC Biolegend 356906 1/50
CD19-PB Biolegend 302224 1/50
Sorting human B cells
CD3-AF647 Biolegend 300416 1/50
CD19-PB Biolegend 302224 1/50
CD27-PC7 Biolegend 302838 1/50
IgD-FITC Biolegend 307808 1/50
Coculture Tfh/B cell
CD3-AF647 Biolegend 300416 1/100
CD19-PB Biolegend 302224 1/100
APCCy7 Biolegend 302816 1/100
CD138-PC7 Biolegend 356505 1/100
Human Tfh cytokine production
IL21-PE Biolegend 513003 1/50
Spleens were stained with Biotin-conjugated anti peanut allergen (anti-PNA, Vector lab, reference 1075-5), Alexa Fluor 647 anti-mouse/human B220 (Biolegend, reference 103226), Alexa Fluor 488 anti-mouse CD4 (Biolegend, reference 100529). Secondary staining was done using Streptavidin-Alexa Fluor 405 conjugate (ThermoFisher, reference S32355). The slides were mounted with ProLong Gold antifade mountant (Thermofisher) and the image acquisition was conducted using a Zeiss LSM 780 confocal microscope.

Validation

The antibodies used in the immunofluorescence studies were validated for this use by the manufacturer
- PNAd https://vectorlabs.com/products/biotinylated-peanut-agglutinin-pna/
- B220-PE https://www.biolegend.com/nl-be/products/alexa-fluor-647-anti-mouse-human-cd45r-b220-antibody-2708?GroupID=GROUP658
- CD4-AF488 https://www.biolegend.com/fr-fr/products/alexa-fluor-488-anti-mouse-cd4-antibody-2697
All antibodies used in cytometry were validated on the species studied for use in cytometry by the manufacturer.

## Animals and other research organisms

Policy information about studies involving animals; ARRIVE guidelines recommended for reporting animal research, and Sex and Gender in Research

Laboratory animals

The animals were housed in a pathogen-free Animal Facility in the Beth Israel Deaconess Medical Center (BIDMC, Center for Life Science). Experimental and control animals were bred together (B6.Camk4fl/fl.dlckCre and B6.lpr.Camk4fl/fl.dlckCre) or in separate cages (other strains). Euthanasia was conducted using CO2 cage following the recommendations from the Institutional Animal Care and use Committee.

| | |
|---|---|
| | For immunization, 8-week-old mice were used. For other B6.lpr mice were bred until 40 weeks and sacrificed.<br>B6.129X1-Camk4tm1Tch/J (Camk4−/−, strain 004994)<br>B6.MRL-Faslpr/J (B6.lpr; strain 000482), were obtained from the Jackson laboratory.<br>B6.Camk4fl/fl.dlckCre mice obtained from previous studies, were crossed with the B6.MRL-Faslpr/J.<br>The SV129/Bl6.Icer/Cremα-/- ice were obtained from Günther Schuetz (Das Deutsche Krebsforschungszentrum, Heidelberg, Germany) and were backcrossed with C57BL/6 mice for over nine generations. |
| Wild animals | No wild animal were used in the study. |
| Reporting on sex | For B6.lpr mice, all mice were female (as reported in the manuscript) since they present a more several phenotype.<br><br>For other experiments, male and female were used (with a balance between groups). |
| Field-collected samples | No field-collected samples in the study. |
| Ethics oversight | The animal study protocol was approved by the BIDMC Institutional Animal Care and use Committee. |

Note that full information on the approval of the study protocol must also be provided in the manuscript.

# Flow Cytometry

## Plots

Confirm that:

☒ The axis labels state the marker and fluorochrome used (e.g. CD4-FITC).

☒ The axis scales are clearly visible. Include numbers along axes only for bottom left plot of group (a 'group' is an analysis of identical markers).

☒ All plots are contour plots with outliers or pseudocolor plots.

☒ A numerical value for number of cells or percentage (with statistics) is provided.

## Methodology

| | |
|---|---|
| Sample preparation | Splenocytes were retrieved after mechanical crushing of the spleen in PBS. The cells were stained in PBS at room temperature 15 minutes. |
| Instrument | Instruments from the BIDMC cytometer core (https://www.bidmc.org/research/core-facilities/flow-cytometry-core/facilities-and-equipment)<br>Analyzer : Beckman Coulter CytoFLEX LX 6 lasers (355 nm (UV), 405 nm (Violet), 488 nm (Blue) 561 nm (Yellow Green), 638 nm (Red), and 808 nm (Infrared) lasers).<br>Cell sorter : Becton Dickinson SORP FACSAria II (5 lasers : 355nm UV, 405nm violet, 488 blue, 561 yellow and 640 red). |
| Software | The data were acquired using Becton Dickinson Diva software, version 6.11 (Cell sorting) or Beckman Coulter CytExpert program, version 2.3.1 (Cytoflex Analyzer).<br>All the data were analyzed using FlowJo 11.8.1 |
| Cell population abundance | The purity of cell sorting were checked using cytometry and was >= 95%. |
| Gating strategy | FSC/SSC gating was made on the main lymphocyte population studied excluding debris. |

☒ Tick this box to confirm that a figure exemplifying the gating strategy is provided in the Supplementary Information.
